# Sex differences in the development of experimental diabetic retinopathy

**DOI:** 10.1038/s41598-024-73279-x

**Published:** 2024-10-01

**Authors:** Ying Chen, Andrea Schlotterer, Jihong Lin, Nadine Dietrich, Thomas Fleming, Stefanie Lanzinger, Reinhard W. Holl, Hans-Peter Hammes

**Affiliations:** 1 Fifth Medical Department, Medical Faculty Mannheim, University of Heidelberg, Mannheim, Germany; 2 Department of Internal Medicine I and Clinical Chemistry, University of Heidelberg, Heidelberg, Germany; 3 Institute of Epidemiology and Medical Biometry, ZIBMT, University of Ulm, Ulm, Germany; 4 German Center for Diabetes Research (DZD), Munich-Neuherberg, Germany

**Keywords:** Sex, Diabetic retinopathy, Estrogen, Vascular damage, Reactive metabolites, Crystallins, Experimental models of disease, Neurodegeneration

## Abstract

This study aimed to characterize the role of female sex in the pathogenesis of diabetic retinopathy. In the retinae of female *Ins2Akita*-diabetic mice (F-IA), ovariectomized female *Ins2Akita*-diabetic mice (F-IA/OVX), male *Ins2Akita*-diabetic mice (M-IA), and female STZ-diabetic mice (F-STZ), the formation of reactive metabolites and post-translational modifications, damage to the neurovascular unit, and expression of cellular stress response genes were analyzed. Compared to the male diabetic retina, the concentrations of the glycation adduct fructosyl-lysine, the Maillard product 3-deoxyglucosone, and the reactive metabolite methylglyoxal were significantly reduced in females. In females, there was also less evidence of diabetic damage to the neurovascular unit, as shown by decreased pericyte loss and reduced microglial activation. In the male diabetic retina, the expression of several members of the crystallin gene family (Cryab, Cryaa, Crybb2, Crybb1, and Cryba4) was increased. Clinical data from type 1 diabetic females showed that premenopausal women had a significantly lower prevalence of diabetic retinopathy compared to postmenopausal women stratified for disease duration and glycemic control. These data emphasize the importance of estradiol in protecting the diabetic retina and highlight the pathogenic relevance of sex in diabetic retinopathy.

## Introduction

Overall, diabetes is more prevalent in men than in women^1^. More specifically, men are more susceptible to diabetes up to the age of 65 years, but women increasingly predominate in the older population^2^. Previous data has suggested that diabetic retinopathy is more prevalent in men than in women, possibly attributable to the effect of testosterone on angiogenesis^3^. The analysis of the relationship between hormones and ocular disease reveal that both, androgen and estrogen receptors are present throughout the eye and these steroids are locally produced in ocular tissue^4^. Since both estrogen receptor subunits ER-alpha and ER-beta are present in the retina^5^, estradiol could exert its vasoprotective^6^ and anti-inflammatory effects^7^ locally.

Gender has a modifying impact on the clinical course of diabetic retinopathy (DR). Several studies report that men have an increased risk of developing retinopathy in both type 1 and type 2 diabetes. In a clinical study of 8784 patients with type 1 diabetes, 27.4% of whom had retinopathy, male gender was associated with a 32.3% increased risk of developing retinopathy over time^8^. Similarly, male gender had an enhanced risk of retinopathy in type 2 diabetes by 11%^9^. In a study with larger cohort of 20.611 type 2 diabetes, 4294 have DR, among them men have a significant higher prevalence of DR compared to women (22% vs 19.3%; *p* < 0.0001)^10^. Sex differences are already present in preclinical stages of retinopathy, at a time when vascular lesions have not yet developed but neuroretinal dysfunction (male vs. female in type 2 diabetes is 29.2% vs. 16.1%) is already measurable by multifocal electroretinogram (mfERG) as early as up to three years before the manifestation of retinopathy^11,12^. Although many clinical evidences advocate that sex hormones protect female from DR progression, there are controversial observations. Kajiwara et al.^13^ observed a high prevalence of proliferative DR in female type 2 diabetes and conclude that female sex is an independent risk factor for DR development. Other studies reported that female sex is not related to the development of DR^14,15^. In order to unravel the mechanism of female DR and prevent or delay the onset of DR in female diabetes, clarification the relationship of female sex in the development of DR is of great importance.

Sex differences in the pathogenesis of diabetic retinopathy have also been observed in experimentally induced diabetic models. Streptozotocin (STZ) induced diabetes leads to an aggravated phenotype in male mice and rats^16^. A worsening effect of androgens^17^ and a protective effect of estrogens^18^ have been proposed a potential cause of these differences. Animal studies using the STZ model of DR have been performed mostly in males, because anecdotal evidence suggests that retinopathy is more severe in males than in females after a certain duration of diabetes^19–22^. However, female animals also often develop milder hyperglycemia such as the *Ins2Akita* mouse, making it difficult to distinguish between glycemia- and hormone-related effects on retinopathy development.

The aim of this study was to investigate the gender effect on diabetic retinopathy in an *Ins2Akita*- ovariectomized mouse model, with intention to create new therapeutic approaches in the context of gender medicine.

## Results

### Estrogen prevents hyperglycemia development

**Fig. 1:**
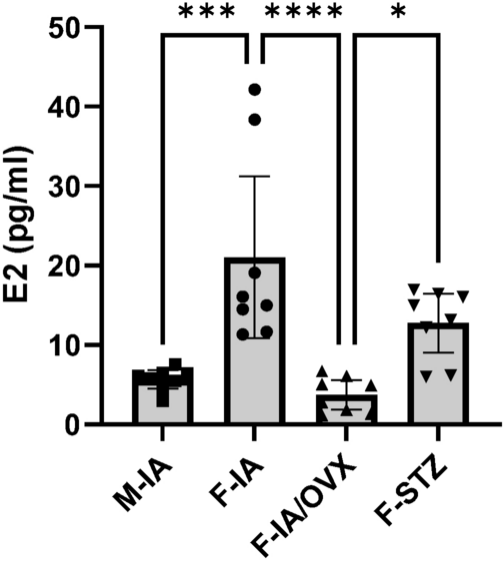
Estradiol levels in experimental groups. The concentration of estradiol (E2) was quantified in serum samples at the end of the study (at age of 26 weeks). M-IA: male *Ins2Akita*-diabetes, F-IA, female *Ins2Akita*-diabetes; F-IA/OVX, ovariectomized female *Ins2Akita*-diabetes; F-STZ, female STZ-diabetes. The data are presented as means ± 95% confidence interval of 8 animals per group. One-way analysis of variance (ANOVA) with Tukey’s multiple comparison test was used to determine statistical differences between groups: *p < 0.05, ***p < 0.001, and ****p < 0.0001.

To determine the difference of the impact of diabetes on estrogen levels as potential determinant of retinopathy development, we measured the serum estradiol (E2) and the blood glucose levels among four experimental groups (M-IA, F-IA, F-IA/OVX and F-STZ, see the group details in the “Experimental groups” section of “Materials and methods”) at the end of the study (26 weeks of age). As expected, ovariectomy (OVX) resulted in significantly lower estradiol levels in F-IA/OVX mice compared to F-IA mice (Fig. 1). STZ-induced diabetes had a marginal insignificant reduction effect on E2 level.

**Fig. 2:**
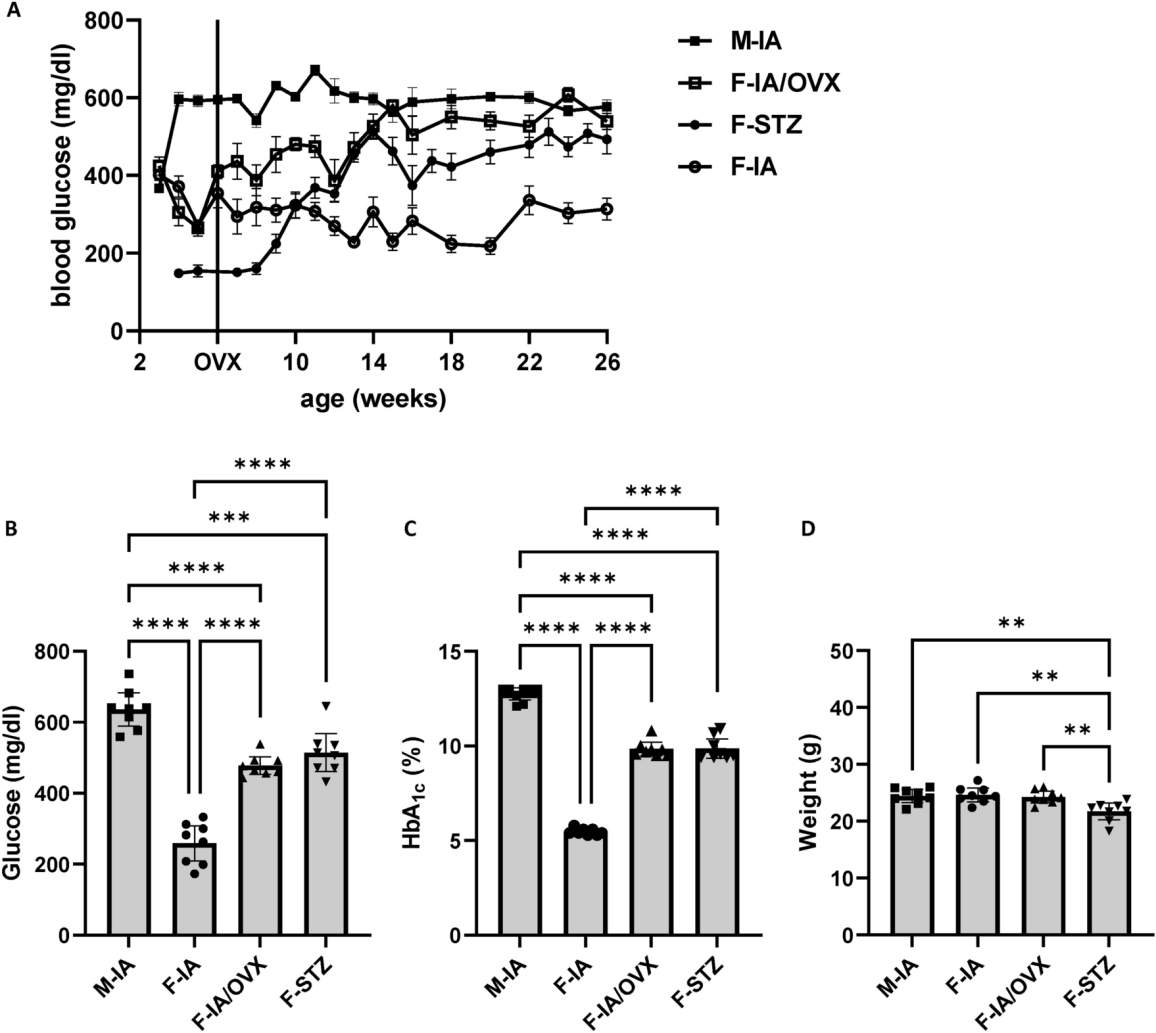
Animal metabolic data of the experimental groups. **(A)** The levels of blood glucose were monitored during the observation period (from age of week 3 to week 26). In addition, (**B**): blood glucose levels, (**C**): HbA_1c_ values and (**D**): body weights were determined at the end of the observation period, at age of 26 weeks. M-IA, male *Ins2Akita*-diabetes; F-IA, female *Ins2Akita*-diabetes; F-IA/OVX, ovariectomized female *Ins2Akita*-diabetes; F-STZ, female STZ-diabetes. In (**A**) The mean values of the blood glucose of 5 animals during the monitored time courses were shown as means ± standard error of the mean. The data in (**B**–**D**) are presented as means ± 95% confidence interval of 8 animals per group. One-way analysis of variance (ANOVA) with Tukey’s multiple comparison test was used to determine statistical differences between groups: **p < 0.01, ***p < 0.001, and ****p < 0.0001.

Noticeably, after OVX, blood glucose levels increased constantly and were similar to males at the end of the study (Fig. 2A), indicating that low level of estradiol cannot prevent hyperglycemia development, namely estradiol protects pancreatic endocrine function.

Compared with the female control group (F-IA), males (M-IA) had significantly higher blood glucose levels from the start (Fig. 2B, C). Therefore, a comparison between female and male animals (F-IA vs. M-IA) was used to identify the influence of glycemia on the development of diabetic retinopathy. Compared with ovariectomized females (F-IA/OVX), female STZ diabetic animals (F-STZ) had significantly higher estradiol levels (Fig. 1) but similar blood glucose levels (Fig. 2B, C). Therefore, comparison of these groups (F-IA/OVX vs. F-STZ) was used to identify estradiol-dependent effects.

### Retinal levels of glycation adducts and reactive metabolites are reduced in females

**Fig. 3:**
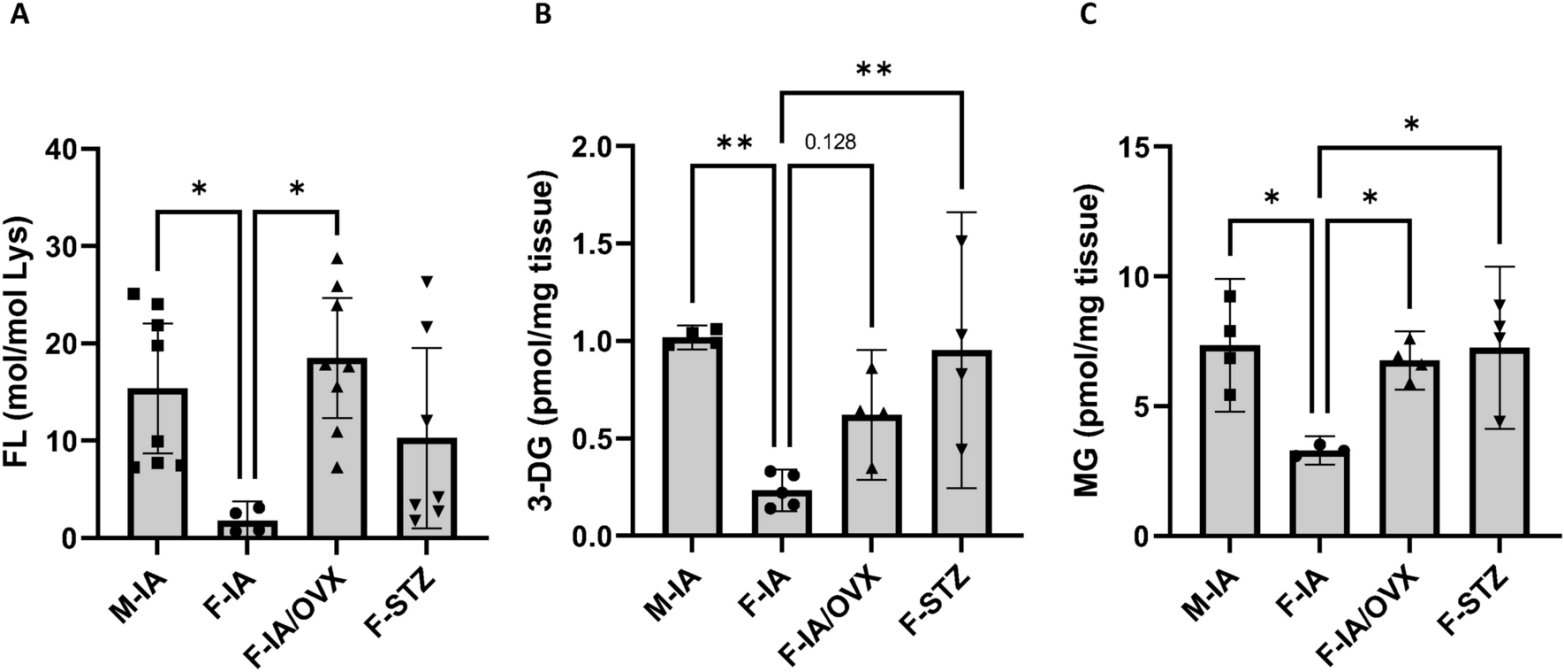
Retinal levels of FL, 3-DG and MG were reduced in females. In the retinal tissues, (**A**) the accumulation of the post-translational modification fructosyl-lysine (FL) and the concentration of the reactive metabolites (**B**) 3-deoxyglucosone (3-DG) and (**C**) methylglyoxal (MG) was quantified. M-IA, male *Ins2Akita*-diabetes; F-IA, female *Ins2Akita*-diabetes; F-IA/OVX, ovariectomized female *Ins2Akita*-diabetes; F-STZ, female STZ-diabetes. The data are presented as means ± 95% confidence interval of 3–8 animals per group. One-way analysis of variance (ANOVA) with Tukey’s multiple comparison test was used to determine statistical differences between groups: *p < 0.05 and **p < 0.01.

Levels of the glycation adduct fructosyl-lysine (FL) were increased in the retinae of ovariectomized females and in the retinae of males compared with the female control group (F-IA) (Fig. 3A). Retinal levels of the Maillard product 3-deoxyglucosone (3-DG) were increased significantly in males (M-IA) and moderately in ovariectomized females compared to females (F-IA) (Fig. 3B). Retinal levels of the reactive metabolite methylglyoxal (MG) were increased in ovariectomized females and in males compared with the female control group (F-IA) (Fig. 3C), suggesting a potential indirect role of E2 in the protection against reactive metabolites. Retinal levels of reactive metabolites (3-DG and MG) in F-STZ mice were comparable to those in M-IA mice and significantly enhanced in comparison to the F-IA group although their E2 levels were similar, which implies that the accumulation of toxic metabolites along with the development of hyperglycemia are dominant in STZ induced female models.

### Early retinal vascular damage of diabetes is protected by E2

**Fig. 4:**
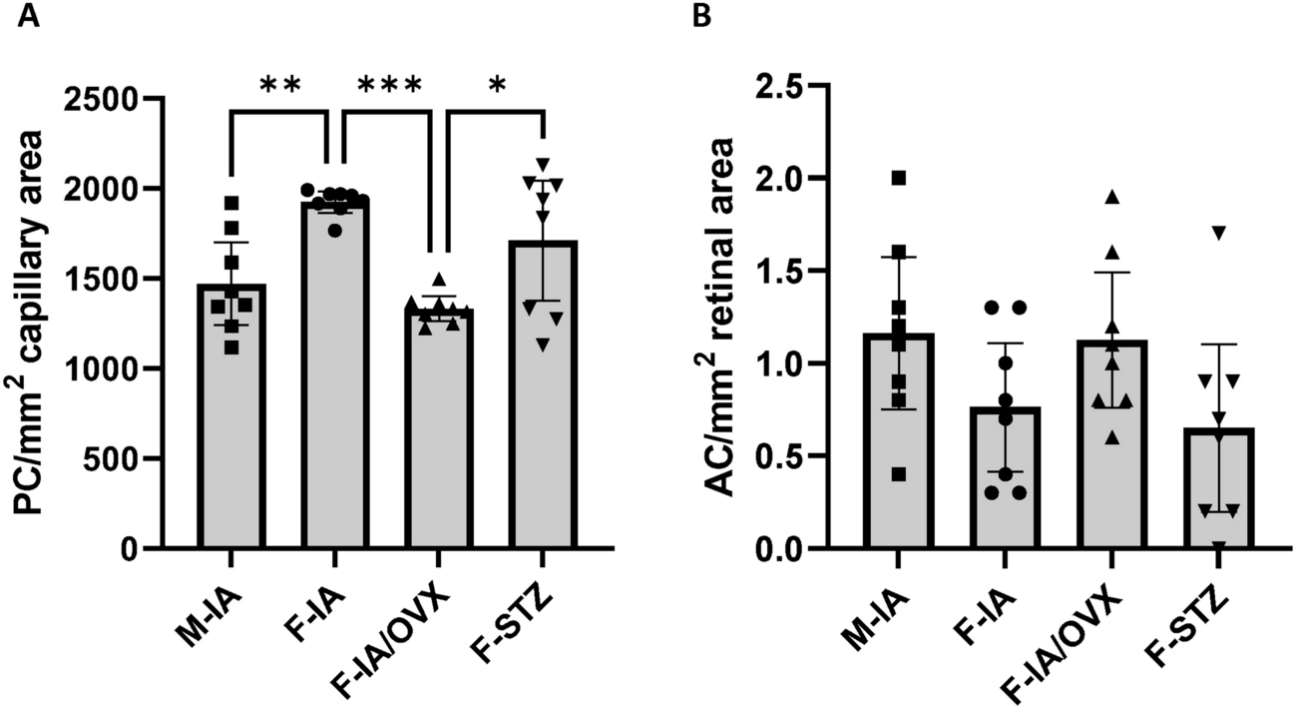
Retinal vascular damage is reduced in females. The extent of vascular damage to the retina was quantified using (**A**) the pericyte (PC) density and (**B**) the occurrence of acellular capillaries (AC). M-IA, male *Ins2Akita*-diabetes; F-IA, female *Ins2Akita*-diabetes; F-IA/OVX, ovariectomized female *Ins2Akita*-diabetes; F-STZ, female STZ-diabetes. The data are presented as means ± 95% confidence interval of 8 animals per group. One-way analysis of variance (ANOVA) with Tukey’s multiple comparison test was used to determine statistical differences between groups: *p < 0.05, **p < 0.01, and ***p < 0.001.

The negative effect of estradiol on the accumulation of glycation adducts and reactive metabolites in the retina was also reflected in decreased damage to the retinal vasculature. At the end of the study, i.e., after 26 weeks of age, female animals with reduced estradiol levels (F-IA/OVX) showed significantly more signs of early vascular damage than female controls (F-IA), as indicated by lower pericyte numbers (Fig. 4A). In addition, we also observed aggravated pericyte loss in females with less estradiol but with similar glucose levels (F-IA/OVX vs. F-STZ). These data suggest E2 has a relevant impact on retinal vascular damage, while glycemia may play a minor role, at least in this experimental context.

The formation of acellular capillaries, an indicator of vascular damage, was slightly increased in F-IA/OVX retina compared to the animals with higher E2 level (F-IA and F-STZ) (Fig. 4B). Although the differences were not statistically significant, it emphasized the E2 protection role in retinal vascular damage from hyperglycemia. The formation of acellular capillaries is often noticed during later periods indicating progressive vascular damage, but variably present during earlier observation periods such as in the present study.

### Microglial activation is restricted at the presence of E2

**Fig. 5:**
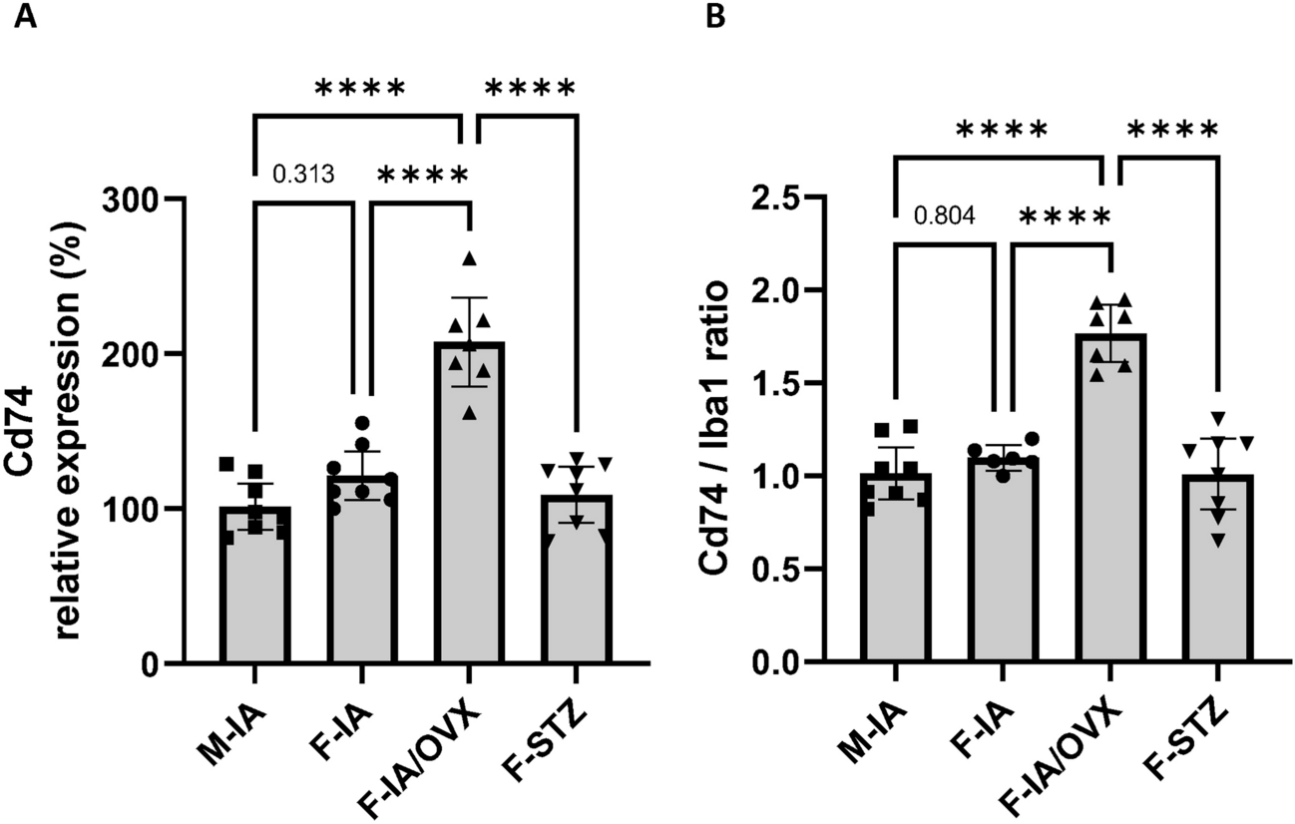
Microglial activation is reduced in females. Microglia activation in the retina was evaluated by the quantification of (**A**) the expression of cluster of differentiation 74 (Cd74) and (**B**) the ratios of Cd74/Iba1 levels. M-IA, male *Ins2Akita*-diabetes; F-IA, female *Ins2Akita*-diabetes; F-IA/OVX, ovariectomized female *Ins2Akita*-diabetes; F-STZ, female STZ-diabetes. The data are presented as means ± 95% confidence interval of 6–8 animals per group. One-way analysis of variance (ANOVA) with Tukey’s multiple comparison test was used to determine statistical differences between groups: ****p < 0.0001.

Microglial activation is considered as a marker of hyperglycemic damage to the retina and may be early involved in the development of diabetic vascular damage^23–25^. The protective effect of estradiol on pericyte loss was accompanied by inhibition of microglial activation in female animals with preserved E2 levels. Compared with female controls (F-IA), the retinae of ovariectomized females (F-IA/OVX) showed a significantly higher number of activated microglia. This was indicated both, by the absolute expression levels of the microglial activation marker cluster of differentiation 74 (Cd74) (Fig. 5A) and by their relative proportions in the expression of the pan-microglial marker Iba1 (Fig. 5B). Similar to pericyte loss, we also observed increased microglial activation in females with reduced estradiol but in similar glucose levels (F-STZ vs. F-IA/OVX). These data suggest that in the present study, the influence of gender on microglial activation is mainly mediated by E2.

### Retinal expression of crystallins is increased in males

**Fig. 6:**
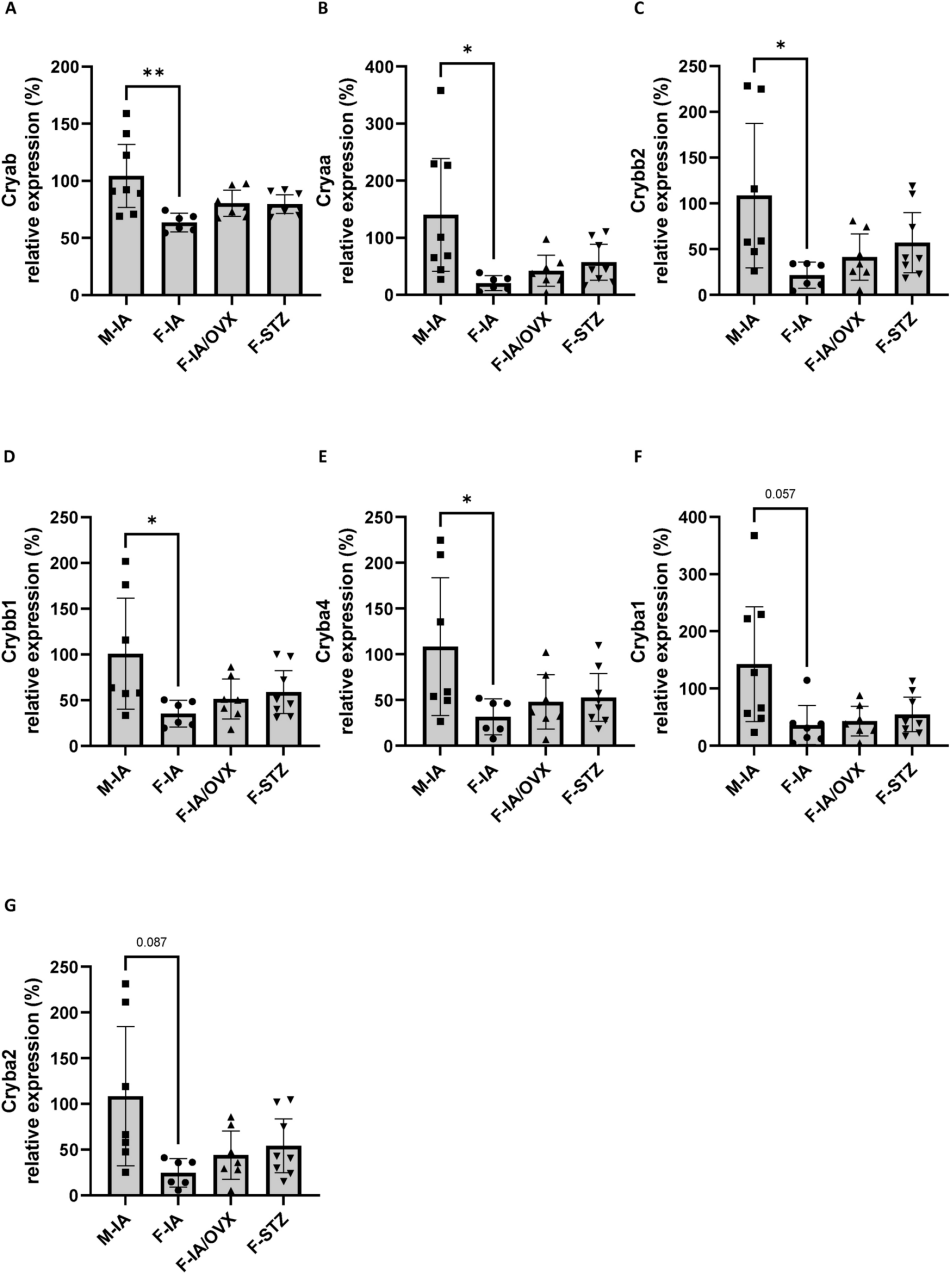
Retinal expression of crystallins is reduced in females. The cellular stress response was measured by the expression of (**A**) crystallin alpha B (Cryab), (**B**) crystallin alpha A (Cryaa), (**C**) crystallin beta B2 (Crybb2), (**D**) crystallin beta B1 (Crybb1), (**E**) crystallin beta A4 (Cryba4), (**F**) crystallin beta A1 (Cryba1), and (**G**) crystallin beta A2 (Cryba2) in retinal tissues. M-IA, male *Ins2Akita*-diabetes; F-IA, female *Ins2Akita*-diabetes; F-IA/OVX, ovariectomized female *Ins2Akita*-diabetes; F-STZ, female STZ-diabetes. The data are presented as means ± 95% confidence interval of 6–8 animals per group. One-way analysis of variance (ANOVA) with Tukey’s multiple comparison test was used to determine statistical differences between groups: *p < 0.05 and **p < 0.01.

Retinal crystallins may act as anti-apoptotic and neuroprotective mediators^26,27^. To identify whether female hormone may influence cellular stress response, retinal expression of several genes of the crystallin family was measured. Male animals exhibited higher retinal levels of certain anti-apoptotic and neuroprotective crystallins. Compared with the female control group (F-IA vs. M-IA), male *Ins2Akita* expressed significantly higher Cryab (45.6%, *p* < 0.01, Fig. 6A), Cryaa (5.8 fold, *p* < 0.01, Fig. 6B), Crybb2 (4.0 fold, *p* < 0.01, Fig. 6C), Crybb1 (1.9 fold, *p* < 0.01, Fig. 6D), Cryba4 (2.4 fold, *p* < 0.01, Fig. 6E), and Cryba1 (2.9 fold, Fig. 6F), and Cryba2 (3.4 fold, Fig. 6G). However, the values among female groups exhibited no differences. These data suggest that retinal crystallin expression might be modulated predominantly by male hormones, e. g. testosterone, as a potential counterregulatory process of protection.

### Clinical data

From the above preclinical results, we hypothesized that estradiol affects the development of diabetic retinopathy in female patients, therefore it is necessary to examine the association between menopause and disease prevalence. For this purpose, pre- and postmenopausal type 1 diabetic women were compared^28^. After adjusting for diabetes duration, BMI, and HbA_1c_, premenopausal women showed a significantly lower prevalence of diabetic retinopathy compared to postmenopausal women (Table 1). The odds ratio for post- versus premenopausal retinopathy, with adjustment for diabetes duration and metabolic control, was 1.56 [95%CI: 1.003–2.429]. This clinical finding supports our hypothesis that the normal level of estradiol protects the diabetic retina from progressive development.

**Table 1:** Prevalence of diabetic retinopathy is reduced in premenopausal women.

Menopausal group	Number of patients	Raw retinopathy prevalence (%)	Adjusted retinopathy prevalence (%)	*p*-value
Post	263	16.34	16.12	
Pre	463	12.52	10.96	*p* = 0.048

The difference in retinopathy prevalence between pre- and post-menopausal women with type 1 diabetes was determined in the regression model after adjustment for diabetes duration and mean HbA_1c_ in the three years prior to retinopathy assessment.

## Discussion

In this study we show that gender has pathogenic relevance in diabetic retinopathy. We demonstrate a clear protection effect of estrogen on two compartments of the neurovascular unit: vascular pericytes and microglia by comparing untreated with ovariectomized female diabetic mice. The clinical data which indicate that estrogen has protective effect on the development of diabetic retinopathy, support the experimental data and underline the translational significance of this study.

The animal models—*Ins2Akita* and STZ mice—included in this study develop chronic hyperglycemia by impairment of proper β-cell function. As diabetes develops during young age in both models, they predominantly mimic type 1 diabetes. *Ins2Akita* model derived from a point mutation in the insulin 2 gene that results in loss of β-cell function, systemic hypoinsulinemia and hyperglycemia, as early as 4 weeks of age^29,30^. Streptozotocin (STZ) is a β-cell-specific toxin that induces irreversible damage to pancreatic islets through free radical generation and DNA damage, which has widely been used to induce diabetes in animals^31,32^. By comparing ovariectomized *Ins2Akita* mice (F-IA/OVX) with female STZ mice, this study design allowed comparison of two groups with the same blood glucose level but different estradiol levels without pharmacological intervention and the potential impact on glucose metabolism.

Consistent with previous animal studies^16,21,33^, our finding confirms that female animals develop milder hyperglycemia. This difference is mainly due to the protective effect of estrogens on beta-cells under metabolic stress^20,34^. Since there is overall agreement that in contrast to other target tissues of diabetic complications, chronic hyperglycemia is the dominant causal factor of diabetic retinopathy^35–37^, the comparison of female mice with equivalent glycemia, but different E2 levels can give insight into the role of E2 in the diabetic retina. In our study, estrogen deficiency appears to be the key determinant of early retinal damage, because animals with higher glucose but preserved E2 (F-STZ) have lower early retinal damage (pericyte loss) than animals with similar glycemia, but lower E2 (F-IA/OVX), while those with lower glycemia and higher E2 (F-IA) are also protected from retinal damage. This modifier role is interesting, yet need further investigations. A mechanistic explanation for the vasoprotective effect of estradiol is provided by a recent study, which shows that E2 specifically inhibits pericyte migration via receptor-mediated signaling^6^. In a previous study, we demonstrate that soluble epoxide hydrolase (sEH) plays important role in initiating pericyte loss and further promoting the progression of diabetic retinopathy^38^. It is reported that neuroprotection of estradiol in ischemia-induced inflammatory response is partially achieved through the suppression of sEH activities^39^. Taken together, we assume that estrogen mediated retinal vascular protection might via the regulation of sEH activity.

Here we identified the potential anti-inflammatory effect of estrogens in the retina from the inhibited activation of microglia in females. This confirms previous studies that estrogen modulates the production of microglial inflammatory mediators via interactions with estrogen receptor beta^7^. The neuroprotection of estrogen in retina may function through the inhibition of sEH activity which further inhibits the microglial activation and prevents retinal vasoregression^40^. Of note, the microglial activation marker CD74 was selectively increased in the ovariectomized group, whereas it remained unchanged in animals with similar levels of estradiol and glucose and in animals with differing levels of estradiol and glucose. However, gender-independent activation of microglia caused by the surgical removal of the ovaries cannot be excluded^41^.

In further support to the concept that E2 partially protects the retina from hyperglycemic damage^42,43^, our study provides evidence that when E2 is reduced to the levels as in males this protection disappears. We found that despite initial differences in blood glucose levels, the extent of retinal vascular damage was similar in males (M-IA) and ovariectomized females (F-IA/OVX). Estradiol, which was similarly decreased in female OVX mice, appears to be a determinant of diabetic vascular damage. The concept that E2 can modulate the clinical course of DR is also supported by our clinical data, according to which more postmenopausal patients with lower E2 levels develop retinopathy compared to premenopausal patients if the duration of diabetes is similar.

Compared to male diabetic animals, the fructosyl-lysine (FL) levels in the retina were significantly reduced in female animals (F-IA). Group comparisons suggested that this difference is at least partially estradiol dependent. The Amadori compound FL, formed from glucose and lysine, is a direct indicator of hyperglycemia in diabetes^44^ and serves as a marker of glucose-mediated protein modification in the retina^45^. However, the influence of estradiol on the accumulation of FL in the retina has not yet been described. Therefore, it is of great importance to consider the FL level as a sex-specific parameter in the pathogenesis of diabetic retinopathy. The reactive metabolite 3-deoxyglucosone (3-DG) is increased in chronic hyperglycemia mainly by the Maillard reaction^46^. Retinal 3-DG levels also showed a trend towards gender differences between the female and male groups. Methylglyoxal (MG) is another reactive metabolite that is increased in patients with diabetes^47^. In the retina, MG is also produced locally by photoreceptors (H.P. Hammes and A. Bierhaus, *unpublished data*) and may therefore be less dependent on blood glucose levels. However, we demonstrated a clear sex-specific difference in retinal MG levels, which could be mediated by estradiol. A direct involvement of estradiol in the detoxification of methylglyoxal has not been described so far. However, aldo–keto reductases (AKR) are known to metabolize steroids^48^ as well as detoxify reactive metabolites^49^. Therefore, it is conceivable that estradiol modulates methylglyoxal detoxification via, for example, the feedback regulation of AKR. Of note, data from animal models have shown that the modulation of glucose and reactive metabolites leads to vascular damage during the progression of diabetic retinopathy^35^.

Along with the observation of estradiol-mediated effects in the female retinae, we also detected certain changes exclusively in male retinae. We found that the specific induction of a cellular stress response in males according to the significantly higher levels of crystallins alpha B (Cryab), alpha A (Cryaa), and beta B2 (Crybb2) in male diabetic retinae. Cryab and Cryaa are small heat shock proteins that are expressed in the eye and, in addition to structural functions in the lens, also they have anti-apoptotic effects in retinal tissues^26,50^. Crystallin beta B2 (Crybb2) has neuroprotective and regenerative effects in the eye^27^. The comparisons between experimental groups showed that retinal crystallin levels were not directly related to blood glucose or estradiol levels. Although the androgen receptors expressed in the retina may be responsible for the induction^4^, we assume that the male retina may have an increased demand for protective mediators. Nevertheless, we cannot exclude that crystallin induction was secondary response to injury.

Although many studies assume that male sex is more prevalent and the estrogen has protection role in DR^10,42,43^, the controversial observations of the gender differences in the clinical manifestations might due to the differences of ethnic origin, the size of the study cohort, the selection bias, and the drug treatment for diabetes. In addition to the previously reported gender differences in the prevalence of retinopathy with clear male predominance^8,9^, we added the natural reduction of estradiol after menopause as an additional paradigm to support the concept of estradiol protection from retinal vascular damage when comparing groups with similar exposure time to diabetes. To the best of our knowledge, similar data have not been reported previously.

In conclusion, we assume that estradiol has a partial protective effect on the diabetic retina. Sex-specific differences in experimental diabetic retinopathy are already evident at the level of the reactive metabolites. Further on, the male diabetic retina induces a cellular stress response, whereas the protective influence of estradiol in the female diabetic retina is related to the vascular and microglial protection in the neurovascular unit.

## Materials and methods

### Animals

The study was conducted in accordance with the Association for Research in Vision and Ophthalmology (ARVO) guidelines for the use of animals in ophthalmic and vision research and approved by the ethics committee of the Regierungspräsidium Karlsruhe, Germany. This study is reported in accordance with the ARRIVE guidelines.

Male and female C57BL/6J-*Ins2Akita* mice (M-IA and F-IA group) and female C57BL/6J wild-type mice were purchased from The Jackson Laboratory (Bar Harbor, ME, USA). Animals were maintained on a 12-h light–dark cycle with free access to food and water.

Blood glucose was weekly measured with the BGStar monitoring system (Sanofi, Paris, France) from the age of 3 weeks to 26 weeks. Glycated hemoglobin (HbA_1c_) was determined by immunoassay-based chemistry technology using the A1CNow^+^ analyzer (PTS Diagnostics, Whitestown, IN, USA). Estradiol (E2) levels were quantified in serum samples by competitive immunoassay using an enzyme-linked immunosorbent assay (DEV9999, Demeditec Diagnostics GmbH, Kiel, Germany). The animals were euthanized by deep anesthesia at age of 26 weeks. Eye and plasma samples were immediately collected and snap-frozen in liquid nitrogen for further analysis.

### Experimental groups

Four experimental groups were involved in this study.

1. F-IA: (female *Ins2Akita*-diabetic mice), an animal strain that develops diabetes due to a point mutation in the insulin gene, were compared with the following three groups.
2. F-IA/OVX (female *Ins2Akita*-diabetic mice after ovariectomy): F-IA mice were ovariectomized (OVX) at 6 weeks of age, i.e. shortly after the onset of diabetic hyperglycemia (4 week of life) but before reaching sexual maturity (8th week of life). This procedure allowed us to largely exclude glycemia and hormone dependent effects. For anesthesia, 120 mg/kg body weight ketamine and 16 mg/kg body weight xylazine were administered intraperitoneally.
3. M-IA (male *Ins2Akita*-diabetic mice): male mice develop diabetes the same as F-IA.
4. F-STZ (female STZ-diabetic mice): 8-week-old female wild-type C57BL/6J mice were induced diabetes by intraperitoneal injection of streptozotocin (STZ, dissolved in sodium citrate buffer; 140 mg/kg body weight; Merck, Darmstadt, Germany), as previously described^51^. Glucose values above 300 mg/dl were considered as hyperglycemia. Stable hyperglycemia was confirmed 7 days after STZ injection, diabetes duration was 18 weeks.

### Reactive metabolites and post-translational modifications

The concentration of reactive metabolites 3-deoxyglucosone (3-DG) and methylglyoxal (MG) and post-translational modification fructosyl-lysine (FL) in retinal tissues was quantified by liquid chromatography coupled tandem mass spectrometry, as previously described^45,52,53^.

### Vascular damage

Vessels were dissected by retinal digestion preparation and vascular damage was examined by quantitative morphometry as previously described^54^. Briefly, eyes were first fixed in 4% formalin for 24 h. Retinae were then dissected under microscope, incubated in water at 37 °C overnight, and then digested in 3% trypsin/Tris–HCl (pH 7.45) at 37 °C for 1–3 h. On microscope slides, the digested proteins and cells were washed away with water drops until the retinal vessels were exposed. The vessels were then dried on a glass slide, stained with Periodic Acid-Schiff (PAS) and Mayer’s hemalum solution, and mounted.

The number of pericytes and acellular capillaries was then quantified using a microscope (Olympus BX51) at 40× magnification in 10 randomly selected fields. Pericytes were identified using Cell^F^ software (Olympus Opticals, Hamburg, Germany), and their number per capillary area (PC/mm^2^ capillary area) was calculated. Acellular capillaries were identified using an integration ocular, and their dimension per retinal area (AC/mm^2^ retinal area) was calculated.

### Gene expression

Total RNA was isolated from retina using TRIzol reagent (Thermo Fischer Scientific, Waltham, MA, USA) and was reverse transcribed into cDNA using the QuantiTect reverse transcription kit (Qiagen, Hilden, Germany). Gene expression was measured by quantitative real-time PCR in the StepOnePlus system (Thermo Fischer Scientific, Waltham, MA, USA). Microglial activation and cellular stress response were examined using the following PCR primers (Thermo Fischer Scientific, Waltham, MA, USA): Cd74 (Mm00658576_m1), Iba1 (Mm00479862_g1), Cryab (Mm00515567_m1), Cryaa (Mm05858657_s1), Crybb2 (Mm02343649_m1), Crybb1 (Mm00517828_m1), Cryba4 (Mm00517516_m1), Cryba1 (Mm00501613_m1), Cryba2 (Mm00517617_m1), Actb (Mm00607939_s1), and Gapdh (Mm99999915_g1). The levels of relative gene expression were calculated using the delta-delta-Ct method^55^. The expression of the housekeeping gene beta-actin (Actb) or glycer-aldehyde-3-phosphate dehydrogenase (Gapdh) was used for normalization.

### Statistical analyses

Data are presented as means together with 95% confidence intervals. Statistical analyses were performed using Prism 9 (GraphPad Software, San Diego, CA, USA). One-way analysis of variance (ANOVA) with Tukey’s multiple comparison test was used to determine statistical differences between groups. *P* values less than 0.05 were considered statistically significant and were reported as follows: *p < 0.05, **p < 0.01, ***p < 0.001, and ****p < 0.0001.

### Clinical data

To compare the prevalence of clinically diagnosed retinopathy between premenopausal and postmenopausal women, data from the German/Austrian DPV registry (March 2022) were used (see http://www.d-p-v.eu). Screening and grading for the presence or absence of diabetic retinopathy was performed by trained ophthalmologists using direct funduscopy in mydriasis according to the guidelines of the German Diabetes Association as previously described^8,9^. Briefly, retinopathy is graded into mild, moderate and severe non-proliferative diabetic retinopathy, in which the key lesions are microaneurysms/dot haemorrhages (mild), venous calibre changes/beading (moderate), and microaneurysms/haemorrhages in four quadrants or venous beadings in two quadrants or intraretinal microvascular abnormalities in one quadrant (severe). Proliferative diabetic retinopathy is defined as neovascularization from the disc or from elsewhere, vitreous haemorrhages or tractional retinal detachment. Retinal examination by binocular biomicroscopy or fundus photography was recorded in a standardized report format. Age at first pathological eye examination was used as the onset of retinopathy and the worst eye determined the patient’s retinopathy level. Female patients with type 1 diabetes, documented daily insulin dose, and available fundus examination were analyzed. To ensure homogeneity of exposure time to the diabetic environment, only subjects with a diabetes duration between 10 and 20 years were included. Age of 35–45 years was defined as premenopausal, and age of 55–65 years as postmenopausal. 463 premenopausal women and 263 postmenopausal women were evaluated. The group of premenopausal women had a mean age of 40.0 years (median = 40.2, lower quartile = 37.5, upper quartile = 42.4), a mean diabetes duration of 15.1 years (median = 15.2, lower quartile = 12.5, upper quartile = 17.5), and a mean HbA_1c_ of 8.0% (median = 7.7, lower quartile = 6.8, upper quartile = 8.9). The group of postmenopausal women had a mean age of 59.8 years (median = 59.6, lower quartile = 57.5, upper quartile = 62.0), a mean diabetes duration of 14.7 years (median = 14.5, lower quartile = 11.9, upper quartile = 17.4), and a mean HbA_1c_ of 7.9% (median = 7.7, lower quartile = 6.9, upper quartile = 8.5). Linear regression models were used to compare total daily insulin dose with adjustment for age, diabetes duration, BMI, and insulin pump use. Logistic regression analyses with retinopathy (binary) as the dependent variable were adjusted for age, diabetes duration, and mean HbA_1c_ in the three years prior to retinopathy assessment. SAS 9.4 (TS1M7) was used on a Windows Server 2021 mainframe.

## Data Availability

The datasets used and/or analyzed during the current study are available from the corresponding author upon reasonable request.
